# Novel Device Therapies for Heart Failure

**DOI:** 10.3390/jcdd10040165

**Published:** 2023-04-11

**Authors:** Deya Alkhatib, Sakiru Isa, Issa Pour-Ghaz, Asra Butt, Omar Al-Taweel, Ifeoma Ugonabo, Neeraja Yedlapati, John Lynn Jefferies

**Affiliations:** 1 Division of Cardiovascular Disease, University of Tennessee Health Science Center, Memphis, TN 38103, USA; 2 Division of Cardiology, University of Nevada Las Vegas School of Medicine, Las Vegas, NV 89154, USA

Heart failure (HF) therapeutics have advanced significantly over the past few years. Despite this, HF continues to place a significant burden on both patients and the healthcare system. This is evidenced by its heavy impact on the mortality and quality of life of the affected patients and their relatives. It also continues to constitute a huge strain on healthcare costs both in terms of expenditures as well as days lost to hospitalization and resultant disability.

Many of the mechanisms underlying HF still lack effective directed medical treatments. In addition, some patients remain symptomatic despite optimal treatment with the current available medications. These factors justify the continued search for devices to treat or supplement the treatment of these patients. In 2016, the United States’ Congress authorized the Breakthrough Devices Program to improve access to innovative devices used to treat life-threatening conditions, including HF. Since then, there has been an exponential increase in the number of devices developed to treat HF [1]. Some of the targets for these devices include valvular abnormalities, the autonomic nervous system, cardiac contractility, and respiratory irregularities.

Cardiac resynchronization therapy has been available for years, but there are specific criteria for its use, including left ventricular ejection fraction (LVEF) of less than 35% and electrocardiographic evidence of ventricular dyssynchrony [2]. Therefore, only about 30% of patients with HF qualify for this treatment. This necessitates the development of more devices with the potential for further reaching clinical benefits.

Cardiac contractility modulation (CCM) devices have been tested in patients with LVEF of 25% to 45% and New York Heart Association (NYHA) class III or IV symptoms. These devices deliver biphasic high-voltage bipolar signals to the right ventricular septum during the absolute refractory period of the cardiac cycle. They are generally safe and yield significant improvements in exercise tolerance and overall quality of life. A study also found up to a 70% reduction in the composite of cardiovascular death and HF hospitalization in appropriately selected patients [3]. In another study, CCM was associated with objective improvements in global longitudinal strain and myocardial mechano-energetic efficiency [4] In addition to increased cardiac contractility, CCM has been shown to contribute to improvement in calcium handling and reverse cardiac remodeling [5]. A recent multi-center pilot study suggests that these benefits may extend to patients with heart failure with preserved ejection fraction (HFpEF) [6].

Abnormalities of the autonomic nervous system contribute significantly to the pathogenesis of HF. Blunting the sympathoadrenal output has been a long-term focus of HF research. Baroreflex activation therapy (BAT) targets this and attempts to eliminate the sympathovagal imbalance in patients with HF [7]. A recent meta-analysis found that BAT is safe and significantly improves the quality-of-life measures, including 6 min walk distance and NYHA classification in patients with heart failure with reduced ejection fraction (HFrEF) [8].

Elevated left atrial pressure, particularly during physical activities, is a pathologic hallmark across all spectrums of HF. It contributes significantly to exercise intolerance in these patients [9]. Inter-atrial shunt devices (IASDs) reduce left atrial pressure by creating an iatrogenic shunt between the left and right atria [10]. Many promising IASDs are currently being evaluated in clinical trials to improve the quality of life of patients with HF, especially those with preserved and mildly reduced LVEF.

Sleep-disordered breathing (SDB), especially central sleep apnea, has been identified in about 50% of patients with HF [11]. SDB is associated with significantly higher mortality and rehospitalization rates among HF patients [12]. Treatment of SDB with continuous positive airway pressure and adaptive servoventilation has generated mixed outcomes thus far [13,14] However, multiple studies have demonstrated the safety and efficacy of phrenic nerve stimulation (PNS) therapy in improving the quality of life of patients with HF, regardless of the LVEF [15]. With these results, PNS was approved for HF patients in the United States. Asymptomatic diaphragmatic stimulation is another modality that is currently being studied, with promising initial data [16].

Mitral valve regurgitation (MR) is not unusual in HF patients, both as a cause of HF and as a consequence of the pathological changes in advanced HF. Many devices have been developed to facilitate transcatheter edge-to-edge repair (TEER) of the mitral valve, but the outcomes for patients with functional MR were initially mixed [17,18]. More encouraging data showed the benefit of TEER in selected patients with functional MR. Some of these novel devices are still being evaluated in ongoing clinical trials with less restrictive inclusion criteria.

Several devices used for edge-to-edge repair and transcatheter replacement of the tricuspid valve are also undergoing clinical trials. They are hypothesized to improve outcomes in HF patients with significant tricuspid regurgitation (TR), as TR is associated with higher rates of mortality and hospitalizations in HF patients [19].

Left ventricular dilation and remodeling have been identified as key contributors to the pathogenesis and poor outcomes of HF. Less invasive ventricular enhancement (LIVE) is a device system designed to address this theory. LIVE consists of a system of anchors that are implanted into the ventricular walls. This is still being studied in clinical trials, but initial data have shown significant improvement in LVEF and HF symptoms [20]. Inadvertent lead malposition is a rare complication of device placement. Multiple studies, including a recent systematic review, have shown good outcomes in patients with this rare complication [21].

Device therapy for HF is a rapidly expanding field with many benefits. It complements the pharmacotherapy of HF, especially in patients who remain symptomatic despite optimal medical therapy. These novel devices largely target pathologic mechanisms with no current effective medical treatment. Most of these devices are beneficial only in patients with HFrEF, necessitating continuous work in this area to find solutions that help different classes/types of HF.

Devices also eliminate the problem of non-adherence to treatment once they are in place. Many of them require minimally invasive procedures for placement and are generally well tolerated by the vast majority of patients. Providers need additional training for these novel devices. The expertise required for successful placement is expected to grow with the increasing deployment of these devices. Physicians are encouraged to discuss device options with their patients, along with the benefits and potential complications, to help them make informed decisions about their treatment.
